# Atropine, Ondansetron, and Ketorolac: Supplemental Management of Amniotic Fluid Embolism

**DOI:** 10.31486/toj.21.0107

**Published:** 2022

**Authors:** Miranda Long, Jane Martin, Joseph Biggio

**Affiliations:** ^1^ Department of Obstetrics and Gynecology, Ochsner Clinic Foundation, New Orleans, LA; ^2^ The University of Queensland Medical School, Ochsner Clinical School, New Orleans, LA

**Keywords:** *Atropine*, *embolism–amniotic fluid*, *ketorolac*, *ondansetron*

## Abstract

**Background:** Amniotic fluid embolism (AFE) is a rare cause of severe maternal morbidity and mortality. No well-studied protocols are available for management of AFE. We present a case of cardiac arrest secondary to presumed AFE and the use of atropine-ondansetron-ketorolac (AOK).

**Case Report:** A 34-year-old gravida 4, para 2012 underwent a repeat cesarean section at 39 weeks of gestation. After delivery of the placenta, hypoxia and hypotension developed, followed by cardiac arrest. Protocols for management of maternal cardiac arrest were followed. Echocardiogram demonstrated right ventricular dilation and hypokinesis. AOK was administrated during prolonged cardiac arrest, and spontaneous circulation returned. The patient was extubated on postoperative day 3 and discharged on postoperative day 10 without neurologic deficits.

**Conclusion:** Management of AFE should include consideration of the addition of AOK to typical guidelines.

## INTRODUCTION

Amniotic fluid embolism (AFE) is a rare cause of severe maternal morbidity and mortality. Risk factors for AFE include advanced maternal age (35 years or older), cesarean or operative delivery, placental abnormalities, and eclampsia.^1,2^ AFE is hypothesized to occur when amniotic fluid enters the maternal circulation, causing massive constriction of the pulmonary vessels from the activation of physiologic mediators such as histamine, endothelin, and leukotrienes. The activation of these substances leads to multiple detrimental physiologic changes resulting in pulmonary artery hypertension, acute cor pulmonale, and eventual complete cardiovascular collapse.^1^ Current guidelines for management of AFE focus on combating acute right heart failure and subsequent cardiogenic shock due to left heart failure.^3^ The use of atropine (1 mg intravenously [IV]), ondansetron (8 mg IV), and ketorolac (30 mg IV) (AOK) as an adjunctive treatment is widely discussed by obstetric providers as a treatment option that should be considered to supplement other treatment modalities. However, very few documented case reports show the utility and success of AOK for treatment of AFE. We present a case of presumed AFE causing immediate postpartum cardiac arrest and the use of the AOK adjunctive protocol. In this case, the patient survived after prolonged cardiac arrest and is neurologically intact.

## CASE REPORT

A 34-year-old gravida 4, para 2012 presented for a scheduled repeat cesarean section and bilateral tubal ligation at 39 weeks of gestation. The patient's pregnancy was complicated by chronic hypertension, but she required no medication during pregnancy. Her medical history was otherwise uncomplicated.

Upon admission, the patient was taken to the operating room for the planned procedure under regional anesthesia. Delivery of the neonate and placenta was uneventful. Approximately 15 minutes after delivery, while the subcutaneous tissue closure was being completed, the patient complained of a feeling of impending doom. Her heart rate decreased to 40/min, oxygen saturation dropped to 44% on room air, and the patient exhibited decorticate posturing. The patient underwent rapid sequence intubation, after which pulseless electrical activity (PEA) was noted. Advanced cardiovascular life support protocol was initiated, and a maternal code blue was activated. Atropine (1 mg IV) and epinephrine (1 mg IV) were administered with subsequent return of spontaneous circulation. Bedside echocardiogram showed right ventricular dilation and hypokinesis with interventricular septum displacement into the left ventricle, resulting in a severely decreased cardiac output from the left ventricle. Milrinone (20 mg in dextrose 5% in water [D5W] 100 mL infusion) was started. A second episode of PEA was encountered. Atropine (1 mg IV), ondansetron (8 mg IV), and ketorolac (30 mg IV) were administered. In addition, a second dose of epinephrine (1 mg IV) was given, and return of spontaneous circulation was achieved. Nine minutes later, a third episode of PEA was noted, and chest compressions resumed. Dobutamine (1,000 mg in D5W 250 mL infusion) was started, and a third dose of epinephrine (1 mg IV) was administered, with resulting return of spontaneous circulation.

The working differential diagnosis at that time included AFE vs massive pulmonary embolus (PE). Although no embolus was noted during the echocardiogram in the proximal pulmonary arteries, PE could not be excluded. Because of significant and repetitive hemodynamic decompensation and after a multidisciplinary discussion, the decision was made to administer alteplase (100 mg IV) as a potential life-saving salvage treatment for a potential PE. A fourth episode of PEA then occurred, and a fourth dose of epinephrine (1 mg IV) was administered in addition to chest compressions. Return of spontaneous circulation was then achieved. The Figure shows the timeline of events. Once return of spontaneous circulation was achieved and sustained, the patient was started on inhaled nitric oxide. She was transferred to the intensive care unit (ICU), intubated, sedated on propofol (10 mg/mL infusion), and administered vasopressor support with norepinephrine (32 mg in D5W 250 mL infusion) and vasopressin (2 units/mL in D5W 100 mL infusion). Massive transfusion protocol was initiated in anticipation of hemorrhage that would ensue after administration of alteplase in the immediate perioperative period. Laboratory workup prior to administration of alteplase demonstrated early disseminated intravascular coagulation (DIC). Repeat laboratory workup after the patient's transfer to the ICU showed fulminant DIC (Table).

**Figure.**
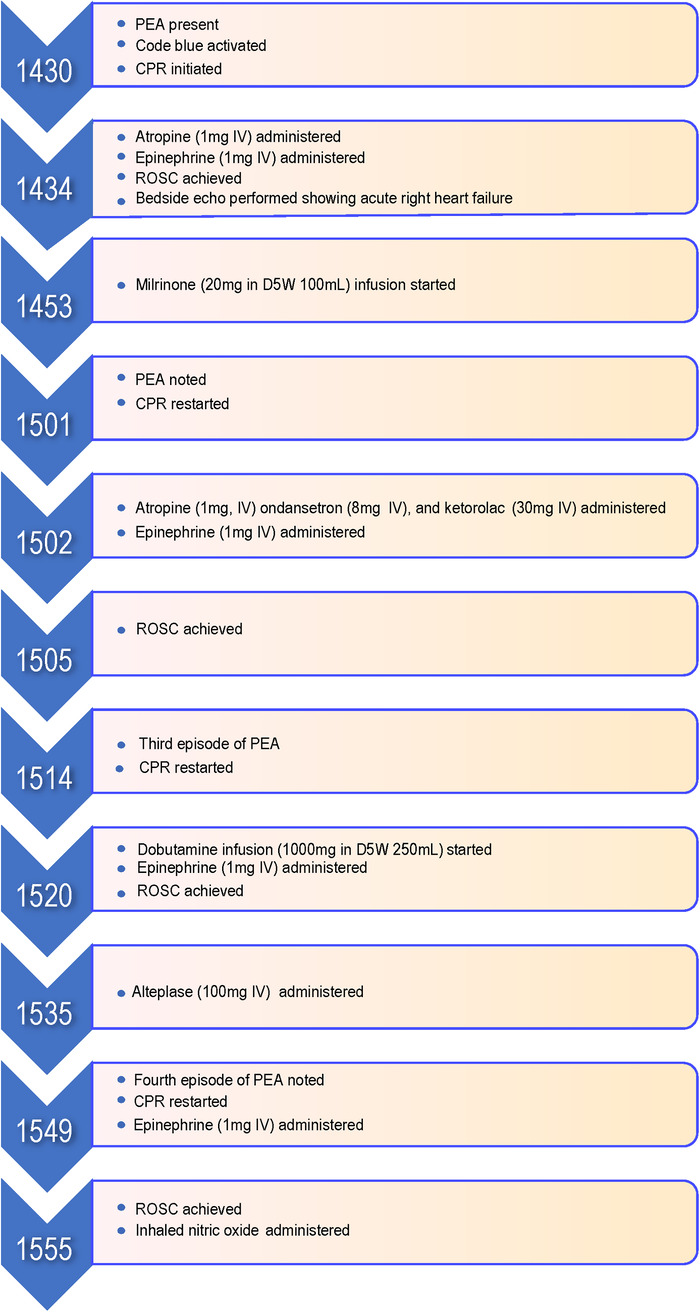
**Timeline of code blue; numbers on arrows correspond to actual times.** CPR, cardiopulmonary resuscitation; D5W, dextrose 5% in water; IV, intravenous; PEA, pulseless electrical activity; ROSC, return of spontaneous circulation.

**Table.** Laboratory Values Indicating Disseminated Intravascular Coagulation

Variable	Initial Laboratory Workup Prior to Alteplase Administration	Repeat Laboratory Workup After Intensive Care Unit Admission
White blood cell count, K/μL	17	11
Hemoglobin, g/dL	9	9
Hematocrit, %	29	30
Platelet count, K/μL	211	77
Prothrombin time, seconds	11.5	17.5
International normalized ratio	1.1	1.6
Partial thromboplastin time, seconds	32.5	86
Fibrinogen, mg/dL	217	80
Lactic acid, mmol/L	5.1	8.0
Troponin, ng/mL	0.045	0.045

While in the ICU, the patient developed intra-abdominal hemorrhage that was presumed to be the bleeding effects of alteplase and/or the coagulopathy associated with DIC. Treatment with 2 doses of 1 mg each of tranexamic acid and blood product replacement with 16 units of packed red blood cells (pRBCs), 10 units of fresh frozen plasma (FFP), 3 units of platelets, and 10 units of cryoprecipitate was unable to achieve hemodynamic stabilization. The patient developed increasing abdominal distension and intra-abdominal pressure that were concerning for abdominal compartment syndrome. The patient was taken back to the operating room 12 hours after the initial event; bleeding was noted along the hysterotomy and at the salpingectomy pedicles. Hemostasis was achieved with suture ligation and application of hemostatic agents. Intraoperatively, the patient received 8 units of pRBCs, 4 units of FFP, 5 units of cryoprecipitate, and 1 unit of platelets. On postoperative day 1, patient remained sedated on propofol and fentanyl, on norepinephrine for vasopressor support, and on milrinone for ionotropic support. On postoperative day 2, the patient was weaned completely off norepinephrine and remained sedated with propofol and fentanyl. On postoperative day 3, the patient no longer required ionotropic support or sedation and was successfully extubated. Her recovery was complicated by unilateral hand ischemia that eventually required amputation of 2 distal phalanges. The etiology of the ischemia remains unclear; potential causes include DIC, vasopressor effect, or hypoperfusion during cardiac arrest. The patient was discharged home on postoperative day 10 neurologically intact with no evidence of detrimental effects after prolonged cardiac arrest.

## DISCUSSION

The diagnosis of AFE is clinical, and diagnostic criteria vary depending on which criteria are used; some experts focus on the clinical picture, while others focus more on laboratory and vital sign abnormalities.^4^ Clark et al created uniform diagnostic criteria for identifying AFE that are endorsed by the Society for Maternal-Fetal Medicine (SMFM) and applied in the United States: (1) sudden onset of cardiovascular collapse or hypotension with oxygen saturation <90%, (2) documentation of overt DIC prior to onset of severe hemorrhage/shock-induced coagulopathy, (3) clinical onset within 30 minutes of delivery of placenta, and (4) absence of fever during labor.^5^ Based on these diagnostic standards, our patient met all criteria for AFE.

AFE causes disruption of the fetal-maternal interface with subsequent passage of amniotic fluid to maternal circulation. An inflammatory response occurs in the lungs, triggering pulmonary vasoconstriction that causes a ventilation/perfusion mismatch, leading to severe hypoxemia.^6^ Right heart strain, dilation of the right ventricle, and displacement of the ventricular septum into the left ventricle then result in decreased cardiac output because of aortic obstruction and myocardial ischemia. In addition, the presence of amniotic fluid is hypothesized to activate the extrinsic pathway of the coagulation cascade, leading to development of a consumptive coagulopathy and ultimately DIC. Management of AFE includes interventions that attempt to counteract these pathologic processes.

Current treatments focus on increasing cardiac output and pulmonary vasodilation, decreasing right heart strain, and providing massive transfusion of blood products.^3^ Dobutamine and inhaled nitric oxide are administered to assist with pulmonary vasodilation. The patient in this case received both dobutamine and nitric oxide during resuscitation. Inhaled prostacyclin could also be given to assist with vasodilation and inhibition of platelet activation. Our patient also received AOK, which is a protocol widely discussed and used for resuscitation during AFE but has limited documentation on its use and success.

A literature review identified a single case report in which AOK was used to treat presumed AFE, although the patient did not meet all 4 diagnostic criteria described above.^7^ In the report published by Rezai et al, the patient was managed with cardiovascular support, blood products, and AOK therapy. The patient experienced a rapid reversal of symptoms immediately after administration of AOK and phenylephrine. Our case differs from the Rezai et al case as our patient met all 4 Clark et al criteria for AFE.^5,7^ In addition, our patient did not have immediate reversal and stabilization after administration of AOK but rather temporary improvement.

The hypothesized mechanism of action of AOK in the setting of AFE stems from additional reduction in pulmonary vascular resistance. Serotonin and thromboxane (increased in maternal circulation during AFE) can cause a synergistic effect, resulting in platelet dysfunction. Serotonin activates receptors in the pulmonary vasculature, resulting in vasoconstriction and entrapment of platelets.^7^ The platelets are then activated by thromboxane that in return recruits more platelets, leading to worsening pulmonary hypertension.^7^ A centrally mediated reduction of peripheral vascular tone by serotonin then leads to cardiovascular collapse.^8^ Ondansetron is believed to modulate serotonin effect, while ketorolac works through nonsteroidal anti-inflammatory properties to decrease production of thromboxane. Atropine counteracts the parasympathetic effects that contribute to bradycardia.^7^

Although this case clinically appeared to mirror the signs and symptoms of AFE, the patient's only known risk factor was cesarean delivery. The authors recognize that a massive PE is a possible diagnosis that must be considered. The pathophysiology of thromboembolic disease in pregnancy follows the Virchow triad of venous stasis, vascular injury, and hypercoagulable state. Venous stasis is present in pregnancy secondary to progesterone-mediated vasodilation. Vascular injury can be present in pregnancy as a result of increased cytokines and blood volume causing injury to vascular endothelium. Pregnancy is a hypercoagulable state because of the increase in procoagulation factors and the decrease in anticoagulation factors.^9^ Presentation of massive PE is clinically similar to that of AFE with profound hypotension and right heart strain. However, our patient was too unstable to undergo further diagnostic imaging to evaluate for a PE. The decision was made to administer thrombolytics to explore all life-saving measures for the most likely differential diagnoses. In Heavner et al, 23 cases of massive PE were reported with administration of thrombolytic agents such as alteplase for the prevention of maternal death.^10^ Our patient demonstrated significant improvement after administration of alteplase.

## CONCLUSION

This case is presented to demonstrate a successful use of the AOK regimen as a supplement to the current guidelines for management of AFE as published by the SMFM. Acknowledging the possible mechanisms of action behind the pathophysiology of AFE is important to understand why a multimodal therapy regimen is used to optimize the outcomes in patients who experience AFE. Further publication of case reports describing the use of AOK and research efforts are needed to better understand the utility and effectiveness of this regimen in management of AFE.

## Notes

*This article meets the Accreditation Council for Graduate Medical Education and the American Board of Medical Specialties Maintenance of Certification competencies for Patient Care and Medical Knowledge.*
